# Antioxidant genes in cancer and metabolic diseases: Focusing on Nrf2, Sestrin, and heme oxygenase 1

**DOI:** 10.7150/ijbs.98846

**Published:** 2024-09-09

**Authors:** Jitendra Shrestha, Khem Raj Limbu, Rashmi Bhandari Chhetri, Keshav Raj Paudel, Philip M. Hansbro, Yoon Sin Oh, Dong Jae Baek, Sung-Hwan Ki, Eun-Young Park

**Affiliations:** 1 College of Pharmacy, Mokpo National University, Jeonnam 58554, Republic of Korea.; 2 Massachusetts General Hospital Cancer Center, Department of Medicine, Harvard Medical School, Boston, MA 02114, USA.; 3 Centre for Inflammation, Centenary Institute and University of Technology Sydney, Faculty of Science, School of Life Sci., Sydney, NSW 2007, Australia.; 4 Department of Food and Nutrition, Eulji University, Seongnam 13135, Republic of Korea.; 5 College of Pharmacy, Chosun University, Gwangju 61451, Republic of Korea.

**Keywords:** nuclear factor (erythroid-derived 2)-like 2, Sestrin, heme oxygenase 1, metabolic diseases, cancer

## Abstract

Reactive oxygen species are involved in the pathogenesis of cancers and metabolic diseases, including diabetes, obesity, and fatty liver disease. Thus, inhibiting the generation of free radicals is a promising strategy to control the onset of metabolic diseases and cancer progression. Various synthetic drugs and natural product-derived compounds that exhibit antioxidant activity have been reported to have a protective effect against a range of metabolic diseases and cancer. This review highlights the development and aggravation of cancer and metabolic diseases due to the imbalance between pro-oxidants and endogenous antioxidant molecules. In addition, we discuss the function of proteins that regulate the production of reactive oxygen species as a strategy to treat metabolic diseases. In particular, we summarize the role of proteins such as nuclear factor-like 2, Sestrin, and heme oxygenase-1, which regulate the expression of various antioxidant genes in metabolic diseases and cancer. We have included recent literature to discuss the latest research on identifying novel signals of antioxidant genes that can control metabolic diseases and cancer.

## 1. Introduction

Oxidative stress arises from an imbalance between the production of reactive oxygen species (ROS) and the cellular antioxidant defense mechanisms 1, 2. ROS, encompassing superoxide anion (O_2_•-), hydrogen peroxide (H_2_O_2_), and hydroxyl radical (•OH), are indispensable byproducts of cellular metabolism but they can exert deleterious effects when their levels exceed physiological thresholds. Moreover, reactive nitrogen species, such as nitric oxide (•NO) and peroxynitrite (ONOO-), and reactive sulfur species, like hydrogen sulfide (H_2_S) and persulfides, play crucial roles in modulating redox homeostasis and contributing to oxidative stress 3.

ROS are generated through endogenous processes, such as mitochondrial respiration and immune responses, as well as exogenous factors including environmental pollutants and radiation 4. Cells possess sophisticated antioxidant defense mechanisms, including both enzymatic (e.g., superoxide dismutase, catalase, and glutathione peroxidase) and non-enzymatic components (e.g., vitamins C and E), to mitigate oxidative stress. Additionally, naturally occurring antioxidant compounds (caffeic acid phenethyl ester, thymoquinone, rutin, catechin, and berberine) are widely used as antioxidant supplements 5-10. Enzymatic antioxidants, including superoxide dismutase, catalase, glutathione peroxidase, and thioredoxin, are pivotal in conferring protection against oxidative stress due to their efficient ROS-scavenging capabilities 11. The expression of antioxidant enzymes is stringently regulated by transcription factors, including nuclear factor (erythroid-derived 2)-like 2 (Nrf2), Sestrins, and heme oxygenase-1 (HO-1) 12-14. Oxidative stress elicits a complex transcriptional response. A plethora of genes, including those involved in antioxidant defense, deoxyribonucleic acid (DNA) repair, and cellular signaling (e.g., NQO1, TXNRD1, PRDX4, TERT, SMUG1, H2AFX, DUSP1, NOS3, TCIRG1, and PFKR), are upregulated in response to ROS. Conversely, other genes (e.g., PFKP, CYR2, and CAT) exhibit attenuated expression under pathological conditions, including cancer 15-17. Beyond transcriptional changes, oxidative stress can also induce epigenetic modifications. These epigenetic modifications, such as DNA methylation and histone post-translational modifications, represent heritable changes in gene expression without altering the underlying DNA sequence. Targeting these epigenetic alterations presents a promising therapeutic strategy for cancer treatment 18.

Oxidative stress plays a pivotal role in the pathogenesis of various diseases, including metabolic, cardiovascular, cancer, and neurodegenerative disorders 19-21. In the cardiovascular system, elevated ROS contribute to the etiology of hypertension, vasoconstriction, arrhythmias, and cardiac remodeling through mechanisms involving nitric oxide depletion, calcium homeostasis perturbation, and hypertrophic signaling cascades 22. Nitric oxide exhibits dichotomous effects, exerting both neuroprotective and neurodegenerative influences, contingent upon its source and downstream signaling pathways. Neuroprotective actions involve the activation of the CREB and protein kinase B (AKT) pathways, while neurodegenerative effects are often mediated by iNOS induction 23. Metabolic diseases, including type 2 diabetes, obesity, hyperlipidemia and non-alcoholic fatty liver disease, are characterized by metabolic dysregulation and elevated oxidative stress. These conditions share common pathological features, such as insulin resistance, chronic low-grade inflammation, and mitochondrial dysfunction, enhanced protein and lipid oxidation, and compromised antioxidant systems, which collectively promote the production of inflammatory cytokines 24. While other metabolic disorders exhibit similar characteristics, this review will focus on these diseases to provide a comprehensive understanding of the role of oxidative stress and antioxidant genes in their pathophysiology.

ROS have a dual role in cancer. ROS can promote tumorigenesis by stimulating cancer cell proliferation, invasion, and progression through interactions with cancer-associated fibroblasts. Additionally, ROS can induce genomic instability and epigenetic alterations, contributing to cancer progression 25. ROS also activate various antioxidant genes within cancer cells as a self-defense mechanism. Conversely, several anticancer therapies exploit this vulnerability by inducing ROS generation, leading to cancer cell death via apoptosis or necrosis 21.

Nrf2, a pivotal transcription factor, is ubiquitously expressed and orchestrates the expression of numerous cytoprotective genes involved in antioxidant defense, detoxification, and redox homeostasis 26, 27. HO-1, a downstream target of Nrf2, catalyzes the rate-limiting step in heme breakdown, yielding biliverdin and carbon monoxide (CO), along with equimolar iron ions 13. Sestrin family proteins are crucial stress-induced proteins regulated by the Nrf2/ antioxidant responsive element (ARE) pathway 28. Sestrin2, conversely, activates Nrf2 by promoting the P62-dependent autophagic pathway through the degradation of the Kelch-like-ECH-associated protein 1 (KEAP1) 29. The intricate interplay between Nrf2, Sestrins, and HO-1 underscores their critical roles in maintaining cellular redox homeostasis and conferring protection against oxidative stress-related pathologies, including metabolic and inflammatory diseases 30-32.

This review aims to elucidate the fundamental regulatory mechanisms of oxidative stress and antioxidant genes, with particular emphasis on Nrf2, Sestrin, and HO-1, in the pathogenesis of metabolic and neoplastic disorders. Through a comprehensive analysis of the regulatory pathways governing these genes, we endeavor to identify potential therapeutic targets and innovative strategies for the prevention and treatment of these complex pathologies. Understanding the multifaceted roles of Nrf2, Sestrin, and HO-1 in metabolic disturbances and cancer progression is imperative for developing novel therapeutic interventions to improve patient outcomes.

## 2. Pathophysiological role of oxidative stress in metabolic disease

**Figure 1:**
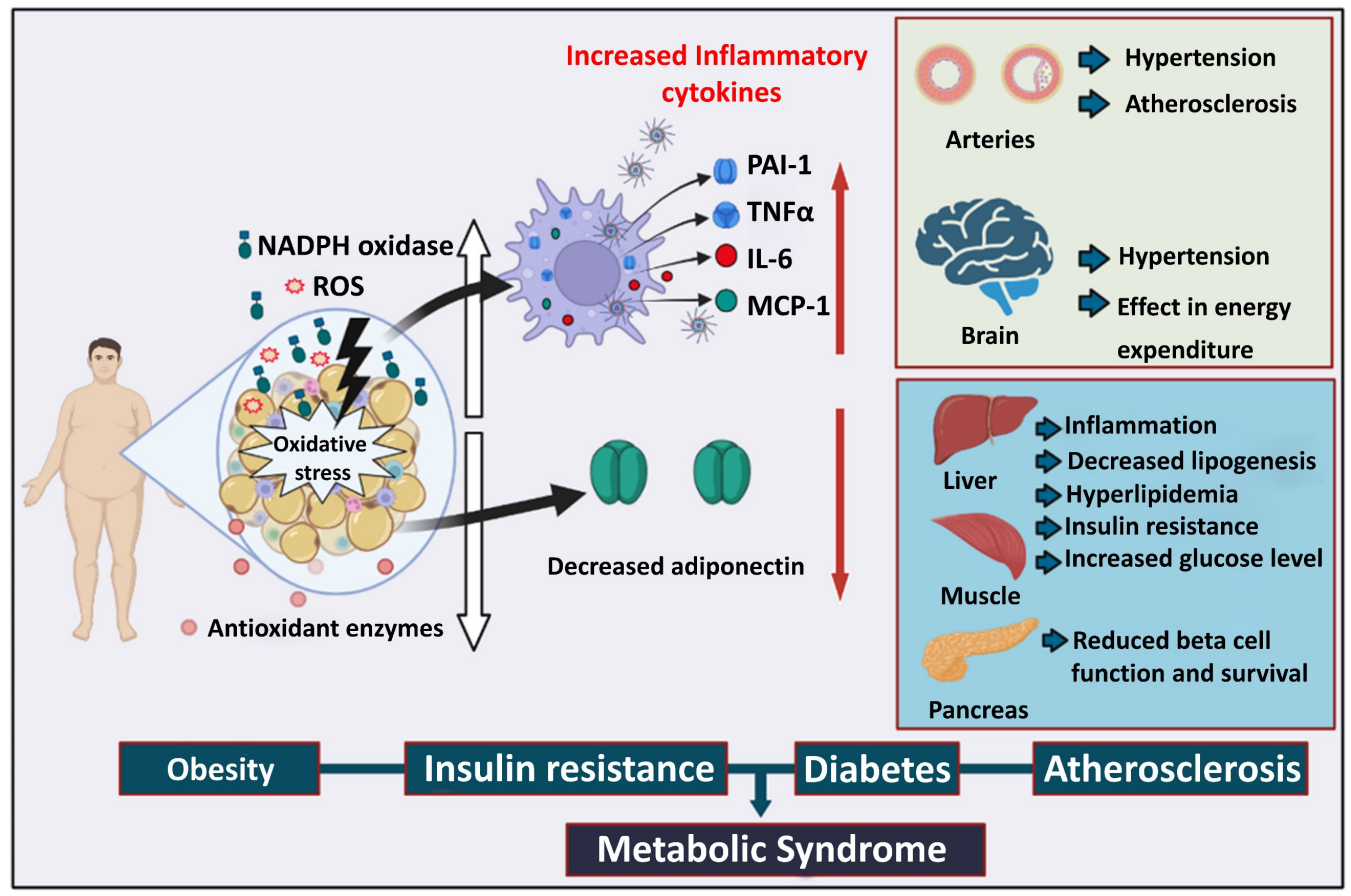
The role of oxidative stress in metabolic syndrome. Obesity promotes the deposition of white fat mass, increasing ROS production, and a reduction in antioxidant enzymes and adiposity. Immune cells exposed to oxidative stress generate a variety of inflammatory cytokines that lead to arterial hypertension and atherosclerosis. Energy expenditure in the brain becomes imbalanced, resulting in brain hypertension. Insulin resistance causes an increase in blood glucose levels, which leads to pancreatic beta-cell malfunctioning and decreases beta-cell survival. ROS: Reactive oxygen species, PAI-1: Plasminogen activator inhibitor 1, TNFα: Tumor necrosis factor alpha, IL-6: Interleukin-6, MCP-1: Monocyte chemoattractant protein-1.

Under oxidative stress, free radicals that are generated in the cell are superoxide anion radical, hydrogen peroxide (H_2_O_2_), nitric oxide, and peroxynitrite 33. The excessive generation of free radicals induces DNA damage, mitochondrial dysfunction, and protein misfolding 33. This cellular damage caused by free radicals ultimately impairs the function of the major metabolic organs 34, 35. Therefore, chronic exposure to ROS inhibits the proper utilization of nutrients, accumulates fat in several organs, and increases blood glucose levels (Figure 1). Oxidative stress is recognized as a cause of metabolic diseases such as obesity, insulin resistance, and diabetes 36 (Figure 1).

Several reports have suggested a close relationship between adipogenesis and ROS levels 37. Nicotinamide adenine dinucleotide phosphate (NADPH) oxidase is important in initiating adipocyte differentiation from pre-adipocytes and mesenchymal stem cells. The knockdown of NADPH oxidase 4 (NOX4), an isoform of NADPH oxidase, inhibited adipocyte differentiation 38, whereas H_2_O_2_-induced oxidative stress and NOX4 overexpression increased lipid storage. The accumulation of ROS in fat due to increased NADPH oxidase causes the inadequate production of adipokines 37. By increasing the expression of NADPH oxidase and decreasing the expression of antioxidant enzymes, the accumulation of fat (for instance, in the adipose tissue of obese mice) caused oxidative stress, which negatively impacted the other organs like the skeletal muscle, liver, and pancreatic beta cells 34. Therefore, excessive oxidative stress appears to be associated with the pathogenesis of obesity-associated metabolic syndrome (Figure 1).

Oxidative stress plays an essential role in the dysregulation of glucose uptake and insulin sensitivity under diabetic conditions 39. H_2_O_2_-induced oxidative stress significantly reduces the mRNA and protein expression of the glucose transporter 4 (GLUT4) in adipocytes and muscles 34. GLUT4 could be involved in peripheral insulin sensitivity and glucose uptake; thus, its expression is important in improving diabetes 40. Oxidative stress impairs insulin signaling by increasing the serine phosphorylation of insulin receptor substrate-1 (IRS-1) or blocking the pathway between IRS-1 and phosphatidylinositol 3-kinase 39. Furthermore, excessive oxidative stress decreases the level of adiponectin, an adipokine that regulates insulin sensitivity and inflammatory response 41. Pancreatic beta cells may be sensitive to ROS because of the low expression of antioxidant enzymes such as CAT and glutathione peroxidase 42. The insulin gene promoter activity and mRNA expression are suppressed in pancreatic beta cells or isolated rat islets when exposed to an oxidative stress inducer 43. Therefore, pancreatic beta cells are important targets for oxidative stress-induced diabetes progression 43. Taken together, excessive oxidative stress plays a critical role in insulin resistance and diabetes progression. Conversely, antioxidant genes appear to exert a protective effect by neutralizing deleterious ROS, thereby protecting pancreatic beta cells from oxidative damage.

Excessive ROS generation leads to oxidative stress, resulting in the development of the chronic inflammatory disease atherosclerosis. Vascular cells under increased oxidative stress experience endothelial dysfunction and more oxidative stress, and they convert low-density lipoprotein (LDL) to oxidized LDL (oxLDL). Macrophages then phagocytose oxLDL, after which they transform into lipid-laden foam cells that release chemokines and cytokines. Lesional macrophages release the inflammatory cytokine interleukin-1β (IL-1β), further exacerbating endothelial dysfunction. This creates a feedback loop that accelerates plaque formation and recruits more monocytes and T lymphocytes into the subendothelial area. These deposits begin as fatty streaks and eventually progress to more intricate forms of atherosclerotic plaque formation 44, 45. Similarly, autophagy protein 5 (ATG5), thiol oxidative stress, 7-hydroperoxide (7-OOH), and macrophage cellular oxidation have been demonstrated to promote macrophage oxidative stress and increase atherosclerotic plaque formation 46. According to recent research, tetrapod PdH nanoenzymes interact with macrophages and strongly inhibit ROS, prevented the oxidative modification of trapped LDL and downregulated TNF-α, IL-1β, and IL-6, thereby exerting a strong antiatherosclerosis effect 47. These findings indicate that increased ROS generation and oxidative stress contribute to the progression of atherosclerosis.

## 3. Pathophysiological role of oxidative stress in cancer

Cancer is one of the most lethal diseases and is characterized by the transformation of aberrant cells harboring diverse mutations in a set of oncogenes that promote their uncontrolled proliferation and dissemination either in local or distant organs, as well as promoting angiogenesis, immortalization, metastasis, and epithelial-mesenchymal transition (EMT) 48. The cellular metabolic rate is considerably upregulated to fulfill the massive energy demands of cancer cells that rapidly multiply. The mitochondrial activity of cancer cells remains intact in most cancer types, although glucose metabolism is enhanced in some cancer types and occasionally converted to anaerobic metabolism, demonstrating Warburg's effect 49. These events in cancer cells ultimately increase the production of various free radicals and ROS within the cancer microenvironment 50. H_2_O_2_ and oxygen free radicals, primarily generated by the mitochondria, are significant ROS in the cancer microenvironment 36, 49.

**Figure 2:**
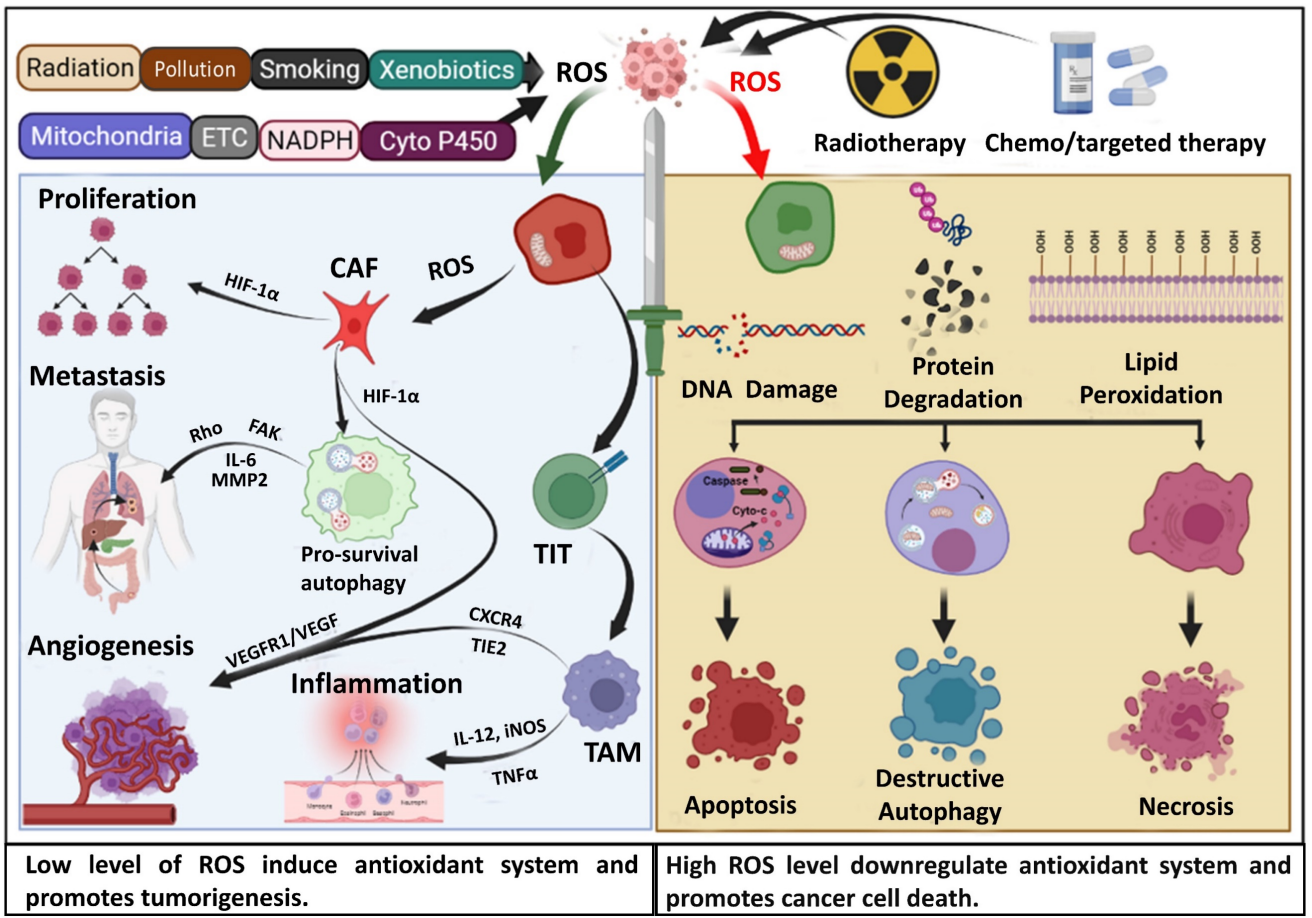
Dual role of oxidative stress and ROS in cancer progression and prognosis. In the complex landscape of cancer, reactive oxygen species (ROS) play a paradoxical role. Originating from a myriad of environmental sources and inherent metabolic processes, ROS, at the early stages of cancer, act as facilitators for tumorigenesis. Beyond this, they further exacerbate the malignancy by promoting cancer progression, particularly enhancing metastasis and advancing angiogenesis. However, this very nature of ROS is harnessed therapeutically with several anticancer chemotherapeutic agents and irradiation techniques. These treatments exploit ROS production to inflict damage on cancer cells, leading to DNA disruption, protein degradation, and lipid peroxidation. This cascade of cellular damage steers the malignant cells towards a variety of fates, including apoptosis, destructive autophagy, and even outright necrosis. ETC: Electron transport chain, CAF: Cancer-associated fibroblast, FAK: Focal adhesion kinase, MMP2: Matrix metalloproteinase-2, TIT: Tumor induced T-cell, TAM: Tumor associated macrophage, HIF-1α: Hypoxia inducible factor 1α, VEGF: Vascular endothelial growth factor, TIE2: The endothelial-specific receptor, tyrosine kinase with immunoglobulin-like loops and epidermal growth factor homology domain-2, CXCR4:C-X-C chemokine receptor type-4.

Considerable literature has described the multifaceted role of oxidative stress in the occurrence of cancer, genetic instability, cancer progression, metastasis, angiogenesis, and cancer treatment by various chemotherapeutic drugs (Figure 2) 21, 51. Perillo et al., 2020 presented ROS as a double-edged sword in cancer in their review, highlighting the role of ROS in cancer progression through the conversion of fibroblasts into cancer-associated fibroblasts (CAF), which then begin to generate ROS and stimulate cancer cell death pathways by the produced ROS in response to various cancer treatment approaches (Figure 2) 21. The ROS generated by CAF stimulates the expression of several antioxidant genes that promote cancer cell growth and invasion 52. Suraweera et al. reviewed the function of antioxidant flavonoids in both the development and treatment of cancer. The function of numerous antioxidant substances has been explored for their specific involvement in cancer, given that the antioxidant system has a dual role in advancing cancer and its treatment 53. Initially, antioxidant activity is regarded as a crucial factor in promoting tumorigenesis. Mutations in Nrf2 and its regulator KEAP1 have been identified in many cancer types 54. The breast cancer susceptibility 1 (BRCA1) gene interacts with and increases Nrf2 expression, thereby improving cancer cell survival 55. In addition, highly efficient peroxiporin activity in pancreatic cancer suggests the presence of an aquaporin AQP5-mediated H_2_O_2_ influx, which activates important cell survival and cancer progression signaling networks. At the same time, AQP3/5 silencing significantly reduced cell migration but was recovered by external treatment with H_2_O_2_ 56. These studies support the idea that antioxidant genes and cancer have a complex functional interaction. Although antioxidant gene expression in normal cells serves to mitigate damage induced by ROS, cancer cells can exploit this system for their benefit 57. First, cancer cells can neutralize ROS produced through their increased metabolic activity or dysfunctional mitochondria. Second, these cells can mitigate ROS generated by anticancer therapies, thereby promoting resistance 58. The precise mechanisms underlying the functional correlation between antioxidant genes and cancer warrant further exploration.

Polychlorinated biphenyls are well-known pollutants that accelerate the advancement of hepatocellular cancer by increasing the pyruvate kinase M2 mediated increment of glycolysis via a ROS-mediated mechanism 59. ROS activates the Distal-less homeobox-2 (Dlx-2)/Snail axis in breast cancer, causing EMT, glycolytic switch, and mitochondrial suppression, which are essential for metastasis. Increased intracellular ROS in colorectal cancer cells caused by fatty acid oxidation stimulates the mitogen-activated protein kinase (MAPK) cascade, causing EMT in ROS high tumor sphere (RH-TS) cells and enhancing metastasis 60. In breast cancer, ROS increased angiogenesis through the extracellular signal regulated kinase 1/2 (ERK1/2)-HIF-1α-VEGF pathway induced by RRM2 and decreased apoptosis by activating the NF-κB pathway 61. The vitagenes gene family encodes antioxidant protein families, including (Hsp) Hsp32, Hsp70, thioredoxin, and sirtuin protein systems. Recent studies have demonstrated the neuroprotective effects of dietary antioxidants, including polyphenols and L-carnitine/acetyl-L-carnitine, through activating hermetic pathways 62. By activating vitagenes, acetylcarnitine can differentially modulate signal transduction cascades, causing apoptosis/cell death in abnormal cancer cells while enhancing defensive enzymes to stop carcinogenesis and neurodegeneration in normal cells 63. This suggests that ROS plays a pivotal role in cancer cell survival, proliferation, EMT, metastasis, and preventing cancer progression at the initial stage (Figure 2).

## 4. The interface of oxidative stress in the relationship between metabolic diseases and cancer

Accumulating evidence suggests that both oxidative and antioxidative genes have functional correlations with metabolic diseases and cancer. Metabolic diseases such as obesity linked to diabetes accelerate the progression of cancer and neoangiogenesis because of increased levels of peripheral estrogens, promitogen cytokines, and growth factors. The long-term inflammation associated with obesity promotes ROS generation, thereby causing cellular damage, stimulating the release of proinflammatory cytokines and transcription factors, and facilitating the spread of cancer 64. According to a recent report, long-term treatment with hypoglycemic drugs such as dipeptidyl peptidase-4 inhibitors in an animal model of breast cancer triggered ROS overproduction and activated the ROS-dependent Nrf2 and HO-1 genes. Nrf2 and HO-1 activation accelerates the metastasis of breast cancer through an oncogenic ROS/Nrf2/HO-1 axis. Moreover, inhibition of the antioxidant gene HO-1 leads to the inhibition of Nrf2 and suppression of breast cancer metastasis-associated genes 65. Elevated ROS levels lead to mutations in mitochondrial DNA and activate the Protein kinase c (PKC) and MAPK pathways, promoting the deregulation of NF-κB and survival of cancer cells. ROS-dependent PKC amplification both affects the insulin receptor substrate and disturbs insulin signaling, contributing to the development of type 2 diabetes mellitus 66. Similarly, recent research linked the etiology of numerous malignancies to obesity. Obesity is associated with both the diagnosis of cancer and an increased risk of cancer-related death because of the advanced disease at the time of diagnosis. Transcriptional regulation downstream of the lipid chaperone fatty acid binding protein (FABP4) is connected to cellular redox, with Nrf2 acting as an upstream regulator. In response to FABP4, Nrf2 promotes cellular proliferation by reducing ROS activity 67. These findings collectively indicate that antioxidant genes are associated with metabolic disease, and they play functional roles in cancer progression.

## 5. Nrf2 as a multifaceted regulator in metabolic diseases and cancer

### 5.1 Nrf2 as a master transcription factor for redox homeostasis

**Figure 3:**
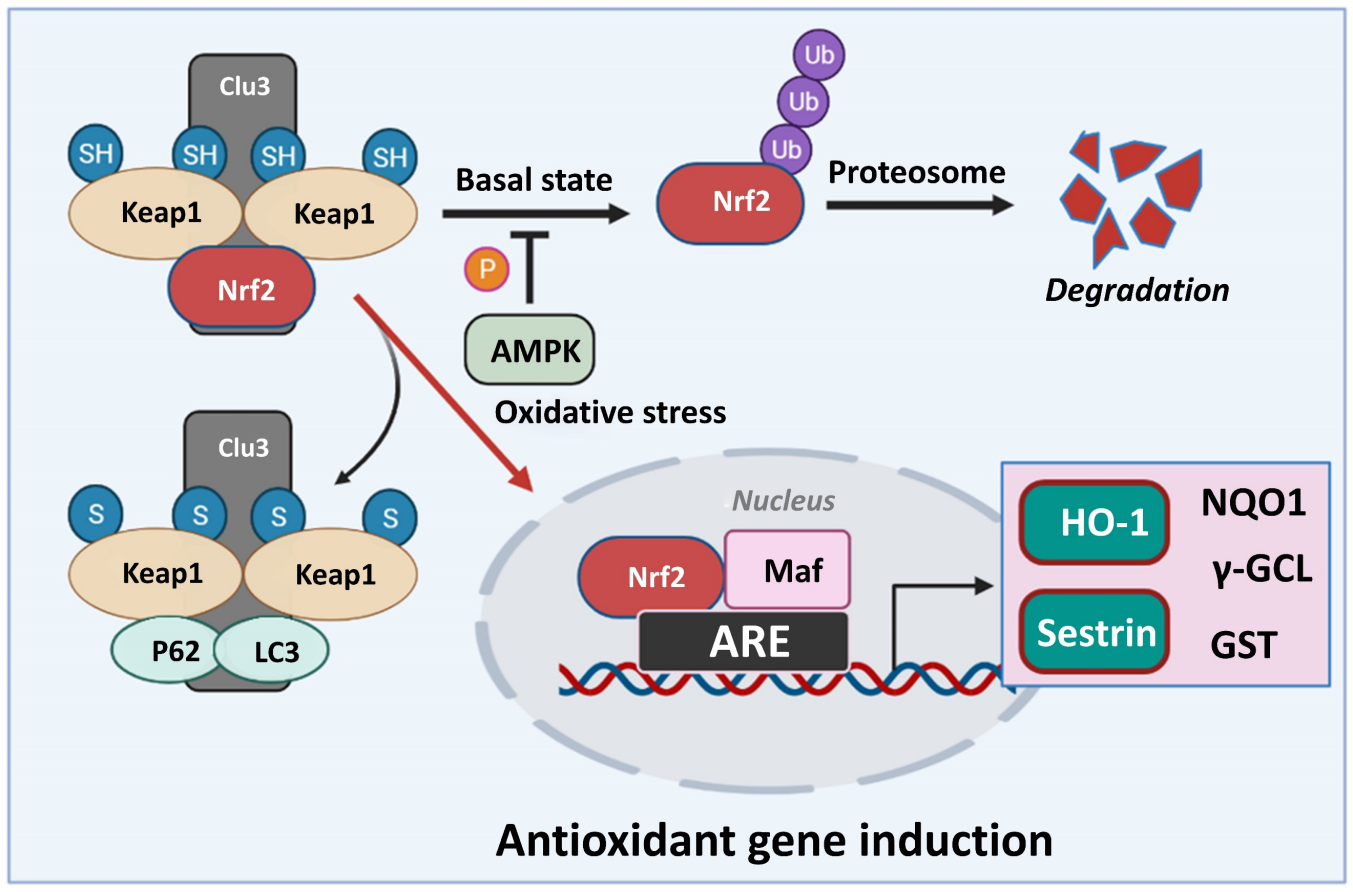
Classical model of Nrf2 activation and response. In basal conditions, Nrf2 remains bound to Keap1, leading to its ubiquitylation and degradation. AMPK phosphorylation regulates the fate of Nrf2 in normal conditions. In response to oxidative stress, Keap1 is inactivated, resulting in Nrf2 stabilization. Nrf2 then translocates to the nucleus, binds to small Maf proteins, and activates the transcription of downstream target genes such as Sestrin, HO-1, NQO1, γ-GCL, GST etc., through antioxidant response elements (AREs). Nrf2: Nuclear factor erythroid 2-related factor 2, NQO1: NAD(P)H quinone oxidoreductase, γ-GCL: gamma-glutamyl cysteine ligase, HO-1: Heme oxygenase-1, GST: Glutathione S-transferase.

Nrf2 is a basic leucine zipper (bZip) transcription factor with a Cap “n” Collar (CNC) structure. Without stress, Nrf2 is retained in the cytoplasm at a low level due to proteasomal degradation 68. The degradation of Nrf2 is regulated by KEAP1, which is a negative regulator of Nrf2. KEAP1 acts as an adapter protein for the Cullin 3 (CUL3)/Rbx1 ubiquitin-ligase and a sensor to activate Nrf2 26, 27. Various stressors or Nrf2 activators alter the cysteine residues in KEAP1. These changes interfere with the binding of KEAP1 and Nrf2, thus allowing Nrf2 to surpass the proteasomal degradation and accumulation. The accumulated Nrf2 translocates to the nucleus and binds to the small Maf proteins to form a heterodimer, which regulates the transcription of various target genes with an ARE in the promoter region 69 (Figure 3).

Nrf2 regulates intracellular antioxidant homeostasis by regulating the expression of various proteins. First, Nrf2 modulates the synthesis of glutathione (GSH) by directly regulating the expression of γ-glutamylcysteine synthetase, which is involved in the rate-limiting step of GSH synthesis 27, 70. Nrf2 also regulates NADPH production and enzymes that mediate the reduction of oxidized GSH 71. Furthermore, Nrf2 induces a protein responsible for detoxifying various substances that are removed from the body 27. In addition, Nrf2 contributes to reducing the level of ROS in the cell by regulating the activity of NADPH oxidase 27. Therefore, the amount of ROS is increased in Nrf2-disrupted cells, which are observed to be more sensitive to oxidative stress.

### 5.2 Role of Nrf2 in metabolic diseases

#### 5.2.1 Nrf2 and mitochondrial function

Mitochondria are one of the primary sites in cells that produce ROS. They are also essential organelles that initiate cell death signaling by regulating membrane potential. Thus, under oxidative stress conditions, mitochondria can be regarded as a source of oxidative stress and an organelle that induces cell death 72. In addition, mitochondria produce adenosine triphosphate (ATP) and the importance of ATP in metabolic diseases is widely recognized 73.

Several studies have reported the role of Nrf2 in mitochondrial function. In Nrf2-KO cells, the amount of produced ROS in the mitochondria is increased 74, which may be due to the destruction of the function of complex 1 in the mitochondrial electron transport system. Nrf2 also affects the mitochondrial membrane potential 75. Decreased mitochondrial membrane potential is important for determining cell death because it is the initiation signal that releases cytochrome *c* into the cytoplasm 76. Nrf2 deficiency lowers mitochondrial membrane potential, and the overexpression of Nrf2 by KEAP1-KO upregulates mitochondrial membrane potential 27. In addition, Nrf2 is deeply involved in mitochondrial biogenesis. Compared to the wild-type, Nrf2-KO mice show reduced mitochondrial DNA in the liver 77. An important factor for mitochondrial biogenesis is nuclear respiratory factor 1 (NRF1), which is regulated by Nrf2 78. The Nrf2 deficiency inhibits the increase in NRF1 mRNA caused by incremental exercise in the mouse muscle 78. Moreover, Piantadosi et al., demonstrated that Nrf2 can bind with the ARE region in the NRF1 promoter 79. Since mitochondrial dysfunction is observed in metabolic diseases, the crosstalk between Nrf2 and mitochondria is considered necessary.

#### 5.2.2 Crosstalk between Nrf2 and AMP-activated protein kinase (AMPK)

The goal of cellular metabolism in cells is the production of ATP. AMPK is an enzyme that regulates energy homeostasis by recognizing the amount of ATP. AMPK plays a positive role in various metabolic diseases by inhibiting the ATP consumption reaction while increasing the ATP-producing response 80. Therefore, crosstalk with the AMPK signal is important for the role of Nrf2 in metabolic diseases. Like Nrf2, AMPK is activated by various intracellular stress conditions. In addition, AMPK activates signals that inhibit inflammation and oxidative stress, similar to those mediated by Nrf2 80. Recently, literature on Nrf2 and AMPK signals has accumulated. It has been shown that AMPK directly phosphorylates the Ser550 residues present in the nuclear export signal of Nrf2, thus increasing the nuclear translocation of Nrf2 81. Similar results demonstrated that the AMPK activator increases the nuclear level of Nrf2 82. Zimmermann et al., 2015 also found that AMPK positively regulates the Nrf2-mediated HO-1 signaling in mouse embryonic fibroblasts 83. In addition, the upregulation of Nrf2 in KEAP1-KO mice increased the phosphorylation of AMPK in the liver and primary hepatocytes 84. Taken together, the AMPK signal, which acts as an intracellular energy regulator, is closely related to Nrf2, and this signal crosstalk is expected to contribute to inhibiting metabolic diseases.

#### 5.2.3 Targeting Nrf2 in metabolic diseases

Various experimental results using animal models and Nrf2 activators suggest the role of Nrf2 in metabolic diseases, including obesity and diabetes 85, 86. Genetic activation of Nrf2 in hypomorphic KEAP1 allele mice fed a high-fat diet ameliorates hepatic gluconeogenesis and lipogenesis, which may be mediated by the activation of AMPK 86. In contrast, Nrf2-null mice fed a high-fat diet exhibited severe steatohepatitis with cirrhosis due to the upregulation of lipogenic genes and downregulation of β-oxidation genes and AMPK levels 85. CDDO-Me (2-cyano-3,12-dioxo-oleana-1,9-dien-28-oic acid methyl ester), a potent inducer of Nrf2, reduces body fat, plasma triglycerides, and fatty acids and lowers blood glucose by improving insulin sensitivity in mice fed a high-fat diet and also had an antidiabetic effect in leptin receptor-deficient mice 87. Although CDDO-Me has been discontinued in Phase III clinical trials, it has significant implications for patients with diabetes 88. In addition, several compounds that positively affect metabolic disease are accompanied by Nrf2 activation 89, 90. Nrf2 inducers contribute to the inhibition of diabetes by protecting the pancreatic beta cells from oxidative stress 91. Apigenin, a naturally occurring compound, was found to bind to Nrf2 and inhibit high-fat diet-induced fatty liver through Nrf2 activation 90. Hidrox, a polyphenol compound derived from organic olives, has been shown to have antioxidant and anti-inflammatory properties in rotenone-induced oxidative stress 92. In addition, it has been reported that Ezetimibe, a drug approved for hypercholesterolemia, inhibits diet-induced non-alcoholic steatohepatitis through Nrf2 activation 89. More disease models are required to be validated and applied to humans, but numerous studies support that the activation of Nrf2 contributes to improving metabolic diseases.

### 5.3 Role of Nrf2 in cancer

#### 5.3.1 Dichotomous functions of Nrf2 in cancer

Nrf2 is recognized for its cytoprotective properties, safeguarding cells against diverse cellular stresses. However, recent research has suggested a more nuanced role for Nrf2 in cancer. Although Nrf2 activation offers a protective advantage in normal cells, its constitutive upregulation in cancer cells appears to promote tumorigenesis and resistance to chemotherapeutic agents. This upregulation is correlated with chemoresistance to drugs such as 5-fluorouracil (5-FU) in colorectal cancer 93. Mutations within the Nrf2 pathway, exemplified by those affecting KEAP1, and alternative activation mechanisms contribute to this observed chemoresistance 94. Somatic mutations in the Nrf2 gene, KEAP1, and the E3 ubiquitin-ligase complex CUL3 have been recognized in many types of cancer, and these mutations are responsible for Nrf2 activation. In addition, Nrf2 activation occurs through alternative splicing of Nrf2 protein lacking a KEAP1-binding site or the overexpression of other proteins that bind with KEAP1. Abnormal Nrf2 activation was found to be responsible for chemoresistance in various cancers, including breast, colorectal, pancreatic, lung, and gastric cancers 95, 96.

Nrf2 activation through diverse mechanisms, such as the interaction between ataxia-telangiectasia group D-associated gene (ATDC) and KEAP1 in pancreatic cancer and the overexpression of progestin and adipo-q receptor family member 4 (PAQR4) in non-small cell lung cancer (NSCLC) cells, promotes cancer progression and treatment resistance 95, 97. Conversely, Nrf2 knockout enhances chemosensitivity in cervical cancer 91. The oncogenic potential of Nrf2 is further highlighted by its association with poor prognosis in glioma, while its deletion in glioma stem cells suppresses tumorigenesis.

Ionizing radiation in prostate cancer can activate Nrf2, leading to its nuclear translocation and the upregulation of antioxidant genes (e.g., GCLC, GCLM, HO1), thereby enhancing radiotherapy resistance by increasing GSH synthesis and suppressing apoptosis 98. Patients with glioma and high Nrf2 expression had poorer prognoses, characterized by shorter disease-free survival and higher tumor grades 99. Conversely, Nrf2 knockout (Nrf2-KO) in glioma stem cells suppressed their proliferation and downregulated the key tumor-promoting factors Sox2, BMI-1, and cyclin E, leading to inhibited tumorigenesis in nude mice 100. Similarly, Nrf2-KO in cervical cancer enhanced chemosensitivity and tumor suppression, highlighting its context-dependent role in cancer progression 101. These findings collectively suggest a role of Nrf2 in cancer; hence, further investigation is required to develop therapeutic strategies.

#### 5.3.2 Targeting Nrf2 in cancer

Despite the context- and cancer-specific roles of Nrf2, various therapeutic approaches target Nrf2 to overcome drug resistance and enhance cancer treatment efficacy. Halofuginone, a febrifugine derivative, has emerged as a potential treatment through inhibiting Nrf2 translation and suppressing its cytoplasmic accumulation, thereby overcoming drug resistance and sensitizing lung cancer cells to chemo- and radiotherapy 102. Similarly, chalcone derivatives such as 4-methoxy-chalcone (4-MC), hesperidin methylchalcone, and neohesperidin dihydrochalcone target Nrf2, reducing the expression of the downstream antioxidant gene NQO1 and promoting ROS generation, thereby sensitizing lung cancer cells to chemotherapy 103. In another study, convallatoxin sensitized lung cancer cells to 5-FU by inhibiting Nrf2 via glycogen synthase kinase-3β (GSK-3β) activation 104.

The PI3K/Akt pathway is another target for Nrf2 modulation. CP-673451, which suppresses Nrf2 transcription through PI3K/Akt inhibition, downregulated platelet-derived growth factor receptorβ (PDGFRβ) and induced apoptosis in NSCLC cells, ultimately sensitizing them to cisplatin 105. Similarly, the combination of xanthohumol and phenethyl isothiocyanate modulated Nrf2 in pancreatic cancer, decreasing cytosolic COX-2 and nuclear STAT-3 to induce apoptosis 106. Ailanthone, a plant extract, also inhibited Nrf2 in pancreatic cancer cell lines, leading to decreased proliferation and viability 107.

Nrf2 knockdown combined with chemotherapy has displayed promise in cervical cancer. Studies reported that Nrf2 knockdown in combination with cisplatin, paclitaxel, doxorubicin, or 5-FU reduces GSH levels and promotes cancer cell apoptosis by modulating mitochondrial BAX 108. Cyanidin-3-O-glucoside, when combined with cisplatin in cervical cancer cells, downregulated Nrf2 and its downstream antioxidant proteins, increasing sensitivity to chemotherapy 109. Artesunate treatment in head and neck cancer effectively inhibited cell growth by enhancing Nrf2 inhibition 110. Interestingly, some flavonoids, such as quercetin and procyanidin B2, were reported to activate Nrf2, potentially preventing procarcinogen-induced ROS generation and DNA damage, suggesting a cancer-preventive role in the early stages 101. Together, these studies highlight the potential of targeting Nrf2 for improved cancer therapy outcomes. Inhibiting Nrf2 can increase cancer cell susceptibility to chemotherapy and radiotherapy. Further research is needed to fully understand the multifaceted role of Nrf2 in different cancer types and develop targeted therapeutic strategies.

## 6. Sestrin as a multifunctional regulator in metabolic disease and cancer

### 6.1 Sestrin family proteins

Sestrin belongs to a highly conserved gene family consisting of three genes, namely *Sestrin1*, *Sestrin2*, and *Sestrin3* in vertebrates 89, 111. Although there is a difference in the expression levels, Sestrins are ubiquitously expressed in several tissues. Sestrin1, named PA26 at the time of identification, was found to be a p53 response gene according to a study on tetracyclin-regulated p53 expression systems 112. The expression level of Sestrin1 is sensitive to genotoxic stress and depends on p53 111. In addition, it is reported that Sestrin1 is induced during serum deficiency, and Forkhead box O (FoxO) proteins are involved in regulating its expression 113. Sestrin2 was first identified as a stress-response gene by microarray analysis under hypoxic conditions and was named Hi95 114. Sestrin2 shows strong homology with Sestrin1 and is the “growth arrest and DNA damage” family PA26. It is regulated as a genotoxic stress response similar to Sestrin1 111. It is reported that the expression of Sestrin2 is regulated by hypoxia-inducible factor-1, p53, or Nrf2, depending on the type of stress stimulus and cells 115, 116. Importantly, overexpression of Sestrin2 inhibited cell death by hypoxia/glucose deprivation or H_2_O_2_ 28, 114, 117. Sestrin3 was found by the *in silico* analysis of Sestrin2 and was shown to share homology with Sestrin1 and Sestrin2 118. Sestrin3 is known to be directly regulated by serum and growth factors, and FoxO3A and FoxO1 were identified as direct regulatory transcription factors 119.

Following the identification of Sestrin, subsequent research demonstrated that its expression increases under stress, offering protection to a wide range of cell types 115, 116. Notably, Sestrin2 has been implicated in maintaining redox homeostasis while concurrently engaging in crosstalk with diverse intracellular signaling pathways 115, 116. Considering the well-established role of Sestrin2 in redox homeostasis and its interactions with intracellular signaling pathways, we reviewed its relevance to metabolic disorders.

### 6.2 Sestrin and redox homeostasis

**Figure 4:**
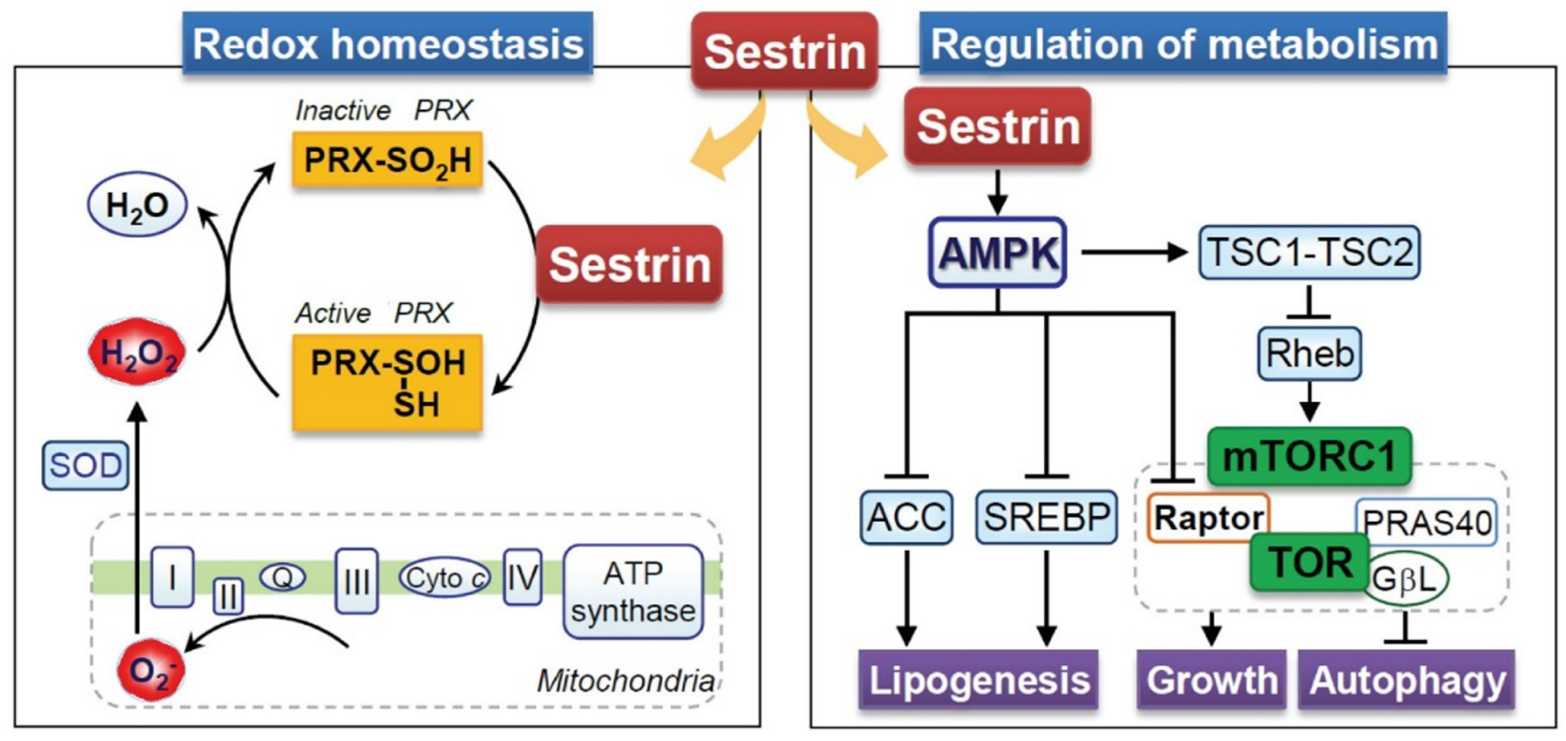
Multifaced role of Sestrin in the regulation of metabolic and cellular redox homeostasis. Sestrin regulates cellular homeostasis over cellular growth, lipid synthesis (lipogenesis), and the vital process of autophagy, mainly by modulating the activity of the mechanistic target of rapamycin complex 1 (mTORC1) and its downstream targets. mTORC1 is a central regulator of cell growth and metabolism, and its dysregulation is implicated in various diseases. This Sestrin-mediated autophagy pathway regulates ROS levels and triggers redox adaptations under stress conditions. To maintain redox homeostasis, Sestrin stabilizes Nrf2 by inhibiting Keap1 and recycling peroxiredoxin (Prx), enhancing antioxidant defense. Activated (Prx) shows antioxidant activity by neutralizing H_2_O_2_ produced in mitochondria. ACC: Acetyl-CoA carboxylase, SREBP: Sterol regulatory element binding protein, SOD: Superoxide dismutase, TSC: Tuberous sclerosis complex.

Sestrin showed low sequence similarity with other proteins at the time of their discovery; therefore, the protein sequence did not contribute to the understanding of the function of Sestrin 115, 116. However, it has been reported that Sestrin contains a conserved region with the prokaryotic protein AhpD, which plays a role in the regeneration of oxidized peroxiredoxin in bacteria 120. In mammals, Sestrin also exerts enzymatic activity by inducing the regeneration of peroxiredoxins (Figure 4). Given the oxidative stress, cysteine residues within peroxiredoxins become excessively oxidized, and thus peroxiredoxins lose their antioxidant activity 121. Reduction of peroxiredoxins by Sestrin re-induces the antioxidant activity of peroxiredoxins 120 (Figure 4). However, research has shown that the regeneration of peroxiredoxins is mainly controlled by sulfiredoxin, which is independent of the Sestrin2 protein 122. Nevertheless, the antioxidant activity of Sestrin has been reported to be beneficial under various stress conditions 28, 117, 123. Sestrin1 and Sestrin2 are known to activate antioxidant signals *via* p53, and the downregulation of p53-mediated antioxidant genes like *Sestrin* contributes significantly to a mutation in p53-suppressive conditions 124. Nrf2-dependent induction of Sestrin2 contributes to suppressing cell death by H_2_O_2_ 28. Conversely, Sestrin2 also activates Nrf2 through the degradation of KEAP1, which plays a vital role in liver protection 29. Moreover, the overexpression of Sestrin2 inhibits methylglyoxal-induced hepatocellular damage at the cellular level and in animal models 125. FoxO induces sestrin3 and contributes to antioxidant activity 119. In addition, Sestrin inhibits ROS in macrophages, thus suppressing the inflammatory response 126. Taken together, it can be concluded that the positive effects of Sestrin on various cells are based on their antioxidant effect.

### 6.3 Sestrin and metabolic homeostasis

The AMPK-mammalian target of the rapamycin (mTOR) pathway is an intracellular energy regulatory signaling pathway that sensitively induces a response in accordance with the redox status 127. This pathway has been recognized to play an essential role in metabolic diseases 80. Sestrin is known to be an upstream regulator of the AMPK-mTOR pathway activity; thus, they are expected to play a positive role in metabolic diseases 80 (Figure 4). Ectopic expression of Sestrin1/2 inhibits the TOR complex 1 (TORC1), one of the two mTOR complexes 111. Sestrin1/2 increases the phosphorylation of AMPK and appears to be due to direct interaction and not its redox activity 111. The activity of TORC1 is regulated by Rheb, a small GTPase, which is negatively regulated by the TSC1:TSC2 complex 128, and AMPK positively regulates the TSC1:TSC2 complex 127. As a result, both AMPK and tuberous sclerosis complex phosphorylation by Sestrin1/2 contributes to the inhibition of mTOR activity 111. Interestingly, Sestrin3 directly activates the mTORC2-Akt signal, which contributes to the enhanced insulin sensitivity of the liver 129. Based on this, Sestrin has positive effects on metabolic disorders. Studies on Drosophila have shown that defects in Sestrin lead to fat accumulation, mitochondrial damage, and muscle degeneration 130. In Sestrin-lacking mice, insulin responsiveness was defective, which promoted high-fat diet-induced insulin resistance and diabetes progression 131. In the muscle cells, Sestrin2 increases autophagy to restore insulin responsiveness through AMPK activation 132. In addition, the overexpression of Sestrin inhibited the Liver X receptor α activity, indicating the potential inhibition of fat accumulation in the liver 133. In nutrient-deficient conditions, Sestrin deficiency makes cells more susceptible to cell death, whereas Sestrin overexpression protects cells from metabolic stress caused by nutrient deficiency 117, 123. Taken together, Sestrin can potentially be a therapeutic target for controlling metabolic disorders.

### 6.4 Role of Sestrin2 in cancer

#### 6.4.1 Multifaceted roles of Sestrin2 in cancer

Sestrin2, a highly conserved protein, plays an important role in response to various types of cellular and environmental stress like DNA damage, hypoxia, oxidative stress, obesity, and endoplasmic reticulum (ER) stress in various tumors and cancers 134. Sestrin2 overexpression is found in various cancers, such as endometrial, colorectal, lung, breast, hepatocellular, bone, and pancreatic cancer, and is involved in the defense mechanisms against cancer. Sestrin2 suppresses cancer cell progression through various signaling pathways. Among them, mTORC1 is one of the key signaling molecules highly expressed in cancer cells and enables the proliferation of cancer 135. The mTORC1 signaling pathway activates the phosphorylation of S6K1 and p40 ribosomal protein kinase s6, inhibiting the translation initiation factor 4E, thereby promoting cancer progression 135. The tumor suppressor gene p53 contributes to the suppression of cancer progression by suppressing mTORC-signaling through the increased expression of Sestrin2 136. In hepatocellular carcinoma, p53 translocation from the cytosol to the nucleus was found to be responsible for Sestrin2 expression, which increased autophagy by activating the Sestrin2-AMPK pathway 137. Sestrin2 reduced colorectal cancer stem cells by inhibiting the Wnt/β-catenin pathway, suggesting that this mechanism may be a potential target for anticancer therapy 138. The upregulation of cellular transformation, tumor progression, and migration occurs through the hyperactivation of the p-Akt/mTORC1/p70S60K pathway, suggesting that Sestrin2, as their regulator, is a potential therapeutic target in cancer 139. In the breast cancer cell line MDA-MB-453ATF4, upregulation of ATF4 upon treatment with Nelfinavir is associated with Sestrin2 expression, which downregulates mTORC1, leading to apoptosis and autophagy 140. Overexpression of Sestrin2 activates AMPK, which inhibits the phosphorylation of mTORC and the proliferating cell nuclear antigen, resulting in the induction of apoptosis (Caspase-3, -7, and -9) in colorectal cancer cell lines SW620 and LoVo, as well as in an *in vivo* mouse model 141. Similarly, in the colorectal cancer cell line HCT116, Sestrin2 overexpression induced apoptosis by inhibiting the mitochondrial membrane potential and generated ROS via the AMPK/p38 signaling pathway 142. In pancreatic cancer, the upregulation of Sestrin2 inhibited the cancer cells' migration, proliferation, and invasion by activating the p-Nrf2/KEAP1/HO-1/NQO-1 signaling axis 143. In human bladder and cervical cancer, Sestrin2 expression enhanced the autophagy of cancer cells by modulating the Sestrin2-dependent MAPK8/JNK1/JUN mechanism 144. In addition, Sestrin2 overexpression inhibits cancer growth by the activation of various signaling pathways such as MAPK4/SAPK/JNK1/Jun kinase, MAPK8/Jun, and the inhibition of x-linked inhibitor protein (XIAP) 145.

According to numerous studies, 146 it has been shown that, in contrast to its role in inhibiting cancer cell growth, Sestrin2 has a progressive role in cancer proliferation, migration, invasion, and chemoresistance. Knockdown of Sestrin2 in the lung cancer cell line A549 suppressed the cancer stemness markers (OCT, SOX2, and Nanog), drug resistance markers, migration markers (Snail, ZEB1, and vimentin), and transcription factors (Nrf2 and HO-1) which indicates that the expression of Sestrin2 upregulated the progression of lung cancer 146. In glutamine-depleted lung cancer cells, Sestrin2 was upregulated, which reprogrammed the lipid metabolism through differential regulation of mTORC1 and mTORC2 and allowed the glutamine-depleted lung cancer cells to survive by maintaining their energy and redox balance 147. Furthermore, Sestrin2 expression in hepatocellular carcinoma was found to be responsible for sorafenib drug resistance via the upregulation of phosphorylation of AKT/AMPKα, leading to carcinoma progression 148. In human squamous cell carcinoma and melanoma cells, UVB radiation increases Sestrin2 levels, thereby enhancing the survival of cancer cells and chemotherapy resistance through the phosphorylation of p-PTEN and phosphorylation of the AKT pathway. Knockdown of Sestrin2 induced chemosensitivity and apoptosis of cancer cells *in vitro/in vivo* 149. In osteosarcoma, Sestrin2 activates autophagy by inhibiting mTOR through the PERK-eIF2α-CHOP pathway, inhibits apoptosis through Bcl-2, and subsequently suppresses the response to anticancer drugs and induces resistance 150. Taken together, these studies suggest that Sestrin2 has both cancer progression and inhibition functions, and further studies of the molecular mechanisms involved can be beneficial for identifying potential therapeutic targets and understanding the physiological properties of cancer.

#### 6.4.2 Targeting Sestrin (Sestrin2 specific) in cancer

Various studies have shown that targeting Sestrin2 represses cancer progression. Among them, one of the anticancer compounds, Fisetin, induces apoptosis through the induction of Sestrin2 via the SESN2/mTORC/Mcl-1 pathway in human head and neck cancer cell lines (MC3, Ca9.22, and HN22) 151. Treatment with Tanshinone IIA in osteosarcoma increased autophagy to prevent cancer cell proliferation and progression by upregulating Sestrin2 via the HGK/SAPK/JNK1/Jun dependent pathway 152. These studies illustrated that Sestrin2 has anti-tumor activity in colorectal, bladder, lung, and endometrial cancer and osteosarcoma by suppressing the mTORC1 signaling pathway. Sestrin2 may be a potential biomarker for tumor repression in these cancer types. Usually, in human colorectal cancer cells, the level of Sestrin2 is found to be lower than in adjacent para-cancerous tissue. However, the cancer therapy treatments oxaliplatin and DHA increase the amount of Sestrin2, obtained when Sestrin2 is bound with CHOP to cause upregulation in colorectal cancer and is strongly associated with ER stress 153. On the other hand, the lentivirus mediated Sestrin2 overexpression significantly enhanced apoptosis in the colorectal cancer cell lines SW620 and LoVo by suppressing the AMPK/mTORC1 pathway 141. Quercetin treatment in HCT116 and HT-29 colon cancer cells increased Sestrin2 expression, thereby activating the AMPKα1 and mTOR pathways and enhancing ROS levels and apoptosis 154. In colon cancer cells treated with 5-FU, Sestrin2 was found to inhibit cell proliferation and migration through a p53-dependent mechanism without increasing ROS levels 155. Moreover, carnosol, a phytochemical compound from rosemary, upregulated Sestrin2 and modulated pERK in HCT116 and SW480 colon cancer cells, thereby promoting the Nrf2/KEAP1 mediated pathway, leading to increased apoptosis and reduced colorectal cancer cell viability 156.

Through the upregulation and stabilization of Sestrin2 mRNA, ChlA-F, a derivative of Cheliensisin A, induces anticancer activity via an autophagy-dependent mechanism to prevent bladder cancer progression 157. Isorhapontigenin treatment effectively increased Sestrin2 by allowing JUN to bind to the AP-1-binding region of the Sestrin2 promoter via MAPK8/JNK1, which subsequently enhanced autophagy by converting LC-3I to LC-3II and increased apoptosis in human bladder cancer cells. In pancreatic cancer, curcumin treatment remarkably repressed pancreatic cancer cell proliferation, migration, and progression through synergistic activity with Sestrin2 via Nrf2/KEAP1/HO-1/NQO-1 143. Various chemical compounds such as 5-FU, excisanin A, curcumin, fisetin, 2-imino-6-methoxy-2H-chromene-3-carbothioamide (IMCA), and quercetin were reported to inhibit cancer cell proliferation through the AMPK/mTORC signaling pathway by upregulating Sestrin2 145, 158. Similarly, IMCA increased the Sestrin2 level in Medullary thyroid cancer, increased the phosphorylation of AMPK, and reduced the phosphorylation of p70S6K, an essential enzyme in the mTOR pathway. This study demonstrated that IMCA drugs function via the p53/Sestrin2/AMPK/mTOR signaling pathway 159. Overall, targeting Sestrin2 in cancer research may be a potential therapeutic strategy to halt cancer growth, invasion, and migration *via* various molecular signaling pathways.

## 7. Role of HO-1 in metabolic diseases and cancer

### 7.1 HO-1 and redox homeostasis

HO-1, a target gene of Nrf2, is a stress-inducible enzyme that catalyzes the degradation of heme into ferrous iron, CO, and biliverdin 160. Two upstream enhancers (E1 and E2) induce the HO-1 gene to maintain redox homeostasis 160. Several AREs are localized in both E1 and E2 regions that play crucial roles in the cellular defense mechanisms against oxidative damage. Oxidative stress promotes Nrf2 translocation and ARE binding activity of HO-1 genes. Besides the redox-dependent transcription factors, BTB and CNC homolog 1 (Bach1), NF-κB, and AP-1 also regulate the HO-1 gene expression under ROS, cytokines, and other pathophysiological stimuli 161. In HO-1-deficient animal and cell models, ROS levels and sensitivity to oxidative stress were increased 162. Evidence has revealed that regulating HO-1 levels plays a role in improving metabolic diseases, such as obesity, insulin resistance, and diabetes 41.

### 7.2 Role and targeting of HO-1 in metabolic diseases

Obesity is one of the causes of metabolic diseases, such as insulin resistance, diabetes, and cardiovascular disease; therefore, its control plays an important role in suppressing various metabolic disorders 35. Several reports have suggested that increased ROS induces pre-adipocyte differentiation and adipogenesis 35, 163. Because oxidative stress plays a critical role in obesity, regulating HO-1 signaling may be the therapeutic target for treating obesity 35, 163. Adipocyte-specific HO-1 overexpression attenuates adiposity and adipogenesis in a diet-induced obesity mouse model. The overexpression of HO-1 in adipocytes increased the reprogramming of pre-adipocytes through the regulation of signaling mechanisms of the paternally expressed gene 1 (Peg1)/mesoderm specific transcript (Mest) and Wnt10b/β-catenin /Sonic hedgehog proteins 164.

The induction of HO-1 by chemical treatment benefits body weight loss and insulin resistance improvement in obese animals 165, 166. Obese mice treated with cobalt protoporphyrin (CoPP), an HO-1 inducer, had a lower body weight and less fat content (visceral and subcutaneous) compared to those in the vehicle-treated obese mice. The increase in HO-1 level reduced adipogenesis in the bone marrow and resulted in a decrease in body weight through the reduction of visceral and subcutaneous fat content 167 (Figure 4). Administration of CoPP in Zucker diabetic fatty (ZDF) rats has been shown to decrease weight gain and fat content 165. In this report, they suggested that the decreased cannabinoid-1 receptor by HO-1 induction may contribute to reducing fat accumulation in ZDF rats 165. Although the exact mechanism has not been elucidated, the administration of hemin, another HO-1 inducer, also reduced body weight and retroperitoneal adiposity in ZDF rats. One possibility is that hemin treatment increased the expression of lipoprotein lipase, an enzyme related to lipolysis, and therefore, it may have enhanced lipolysis in ZDF rats 166. Treatment with butein (flavonoid chalcone) increases HO-1 levels through the p38 MAPK/Nrf2 pathway and reduces adipocyte hypertrophy in mice fed a high-fat diet 168.

Obesity causes adipose tissue hypertrophy, leading to the secretion of various inflammatory cytokines and adipokines, such as TNF-α, IL-1β, resistin, leptin, and adiponectin. This increased protein release regulates insulin resistance and sensitivity 169, 170. In obese diabetic mice, administration of CoPP induced HO-1 protein levels, resulting in increased insulin sensitivity and improved glucose tolerance. The mechanism of action of CoPP could be multifaceted and attributable to the activation of the HO-1-adiponectin axis 167. Insulin sensitivity and obesity are accompanied by chronic inflammation and pro-inflammatory cytokines, produced by the infiltrating macrophages in the adipose tissue 171. It has been shown that adiponectin decreases the generation of pro-inflammatory risk factors.

**Figure 5:**
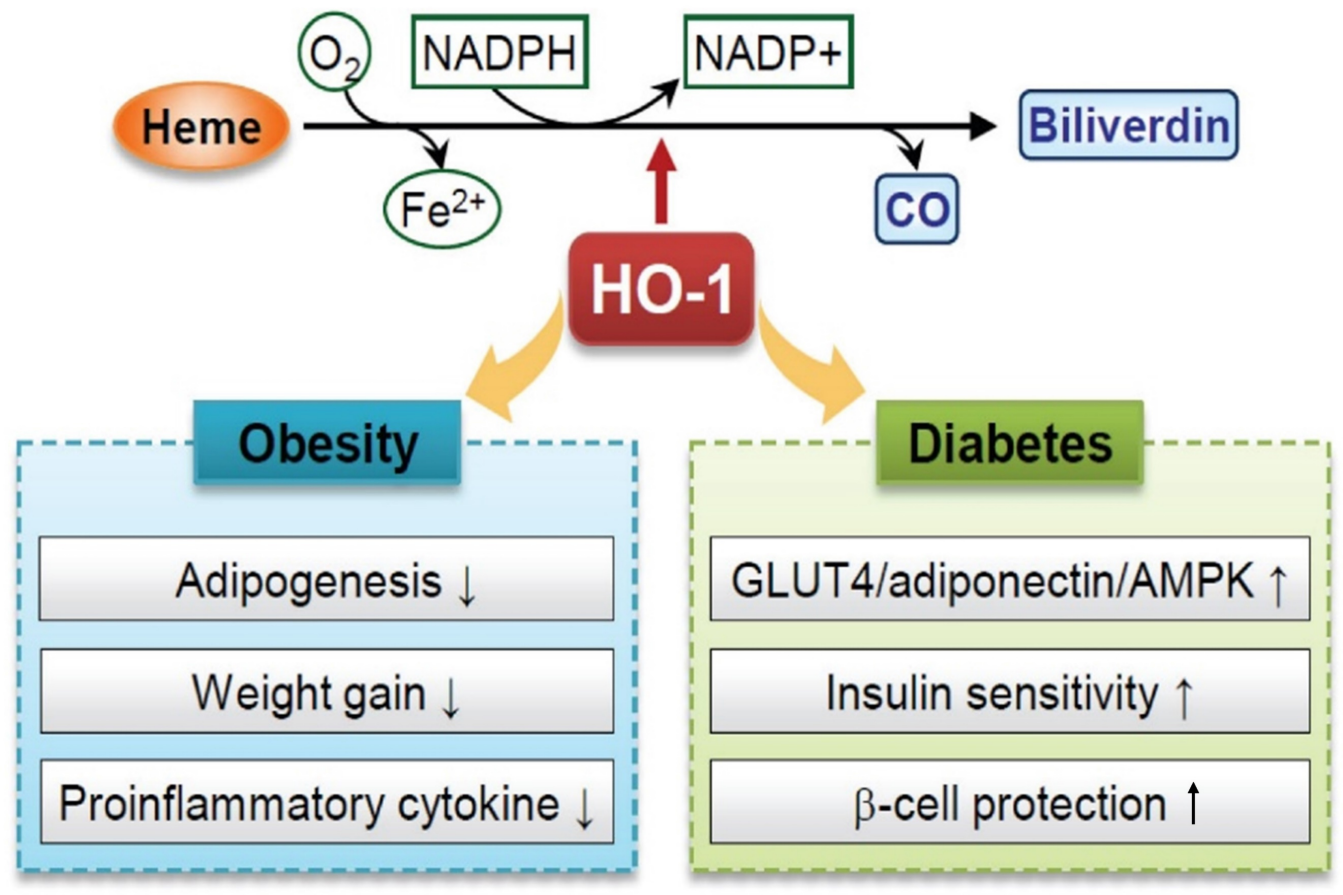
Role of heme oxygenase-1 (HO-1) as antioxidant protein in obesity and diabetes. HO-1 catalyzes the enzymatic breakdown of stress-inducing heme, producing biliverdin/bilirubin, carbon monoxide (CO), and free iron. Biliverdin/bilirubin, through its effects on hypertrophic adipocyte remodeling and inhibition of reactive oxygen species (ROS)-mediated adipogenesis, demonstrates an attenuating effect on adiposity. CO exhibits anti-inflammatory and anti-apoptotic properties, contributing to cardioprotection by inhibiting vasoconstrictive processes. HO-1 also plays a pivotal role in safeguarding vascular function against the deleterious impact of free iron. In the context of prediabetic obesity, HO-1 displays significant enhancements in the functionality of glucose transporter-4 (GLUT-4), adiponectin, and AMP-activated protein kinase (AMPK), promoting insulin sensitivity and beta-cell protection.

Type 2 diabetes mellitus is a chronic metabolic disorder characterized by insulin resistance and hyperglycemia. Various pathologies, including defective insulin secretion from pancreatic beta cells, inadequate hepatic glucose production, and peripheral insulin resistance, are involved in the development of the disease, and several therapeutic approaches have been attempted to treat this metabolic syndrome 172. The expression of HO-1 was reduced in patients with type 2 diabetes, which is associated with insulin sensitivity and oxidative capacity 173, 174 (Figure 5). The upregulation of HO-1 exerts an antidiabetic effect, and the underlying mechanisms include the induction of insulin sensitization and glucose metabolism-related genes, such as GLUT4, adiponectin, AMPK, cAMP, and cGMP. Although the mechanism is not fully understood, treatment with hemin, an HO-1 inducer, increased the adiponectin levels through AMPK signaling activation, and this pathway stimulated the translocation of GLUT4 in Goto-Kakizaki rats, which are non-obese, type 2 diabetic model rats 166.

Excess oxidative stress induces damage to cellular DNA and proteins and causes pancreatic beta-cell dysfunction and death during the progression of type 2 diabetes 175. Studies reported that the HO-1 signaling pathway partially protects beta-cells from palmitic acid-induced lipotoxicity that causes oxidative or ER stress 176. Nrf2 activation or Bach1 deficiency is involved in regulating intracellular oxidative defense capacity through HO-1 induction 176. Taken together, HO-1 can potentially be a suitable target for treating obesity, insulin resistance, and diabetes (Figure 5).

### 7.3 Role of HO-1 in cancer

#### 7.3.1 Functions of HO-1 in cancer

The importance of HO-1 in cancer progression has been highlighted by numerous studies, and its expression is linked to tumor growth, invasiveness, metastasis, angiogenesis, resistance to therapy, tumor immune escape, and poor prognosis through various mechanisms 177. HO-1 promotes aggressiveness and therapy resistance, resulting in a higher risk of cancer development and therapeutic failure because HO-1 accelerates the production of tumor neovasculature and confers a selective advantage in overcoming increased oxidative stress during carcinogenesis and therapy to tumor cells 178. Several studies have suggested that HO-1 is involved in tumor induction and can promote tumor growth and metastasis more effectively through catabolites. HO-1 activity provides antioxidant, anti-apoptotic, and cytoprotective properties and removes harmful intracellular heme from the cell intrinsically and promotes anti-inflammation and immune suppression by modulating the tumor microenvironment when the degradation of heme produces catabolites that diffuse out of the cell 179. In a carcinogenic mouse model and cell line of head and neck cancer, cytoplasmic HO-1 was expressed in the pre-neoplastic lesions and nuclear HO-1 was expressed in tumor tissues. This suggests that the increased expression of HO-1 in the nucleus is associated with tumor growth and may have pro-tumor functions 180. HO-1 upregulation in melanoma reduced the survival of tumor-bearing mice because melanoma cells develop more condensed tumors with increased viability, proliferation, and metastasis 181. HL-60R, a drug-resistant acute myeloid leukemia cell line that overexpresses HO-1, showed less sensitivity to cytarabine and daunorubicin than HL-60 cells. In addition, the downregulation of HO-1 increased sensitivity to chemotherapy in the HL-60R cell line, which indicates that the increased expression of HO-1 correlates with chemoresistance in hematological malignancies 182. Similarly, high HO-1 expression induced IL-6, one of the most important survival factors of cancer cells in multiple myeloma, and increased resistance to lenalidomide treatment 183. Furthermore, HO-1 promoted lung metastasis by inducing the activation of vascular endothelial growth factor (VEGF) and IL-10 184. In a study utilizing colon cancer cells, it was reported that lowering the level of glucose-regulated protein 78 (GPR78) increased the metastatic capacity through the activation of Nrf2/HO-1 signaling and the upregulation of vimentin and downregulation of E-cadherin levels 185. HO-1 also has an important role in modulating immune responses 186. In a study with colorectal cancer cells, HO-1 expression decreased the cell-mediated cytotoxicity by lowering the expression of ICAM-1 and CXCL10, which suppressed the adhesion of peripheral blood mononuclear lymphocytes to the colorectal carcinoma cells and the recruitment of T effector cells which regulate anti-tumor immunity 187. The upregulation of HO-1 activated the Sonic Hedgehog (SHH) signaling pathway. It was found to induce cell proliferation, while the downregulation of HO-1 resulted in decreased cell proliferation in pancreatic cancer (PC) 188

However, in cancer studies, the role of HO-1 is controversial, as several studies have reported that the activation of HO-1 prevented the proliferation of breast cancer 189 and angiogenesis in prostate cancer 190. The overexpression of HO-1 altered the levels of matrix metalloproteinase and oncogenic miR-378 within the tumor microenvironment. It contributed to the downregulation of metastasis, cell proliferation, and migration in mucoepidermoid carcinoma of the lung 191. According to reports by Gandini et al. 2019, HO-1 was found to be related to anti-tumor activity in cell lines and animal and human tumor samples of breast cancer, and the dual effect of HO-1 anti- or pro-tumor activity may be dependent on the subcellular localization of HO-1 192. When colon cancer progressed, upregulation of HO-1 was associated with an increase in the survival time of patients, and this effect is due to HO-1 suppressing the survival and migration of colon cancer cells in a p53 tumor suppressor protein-dependent manner 193. In colorectal cancer, silencing the HO-1 gene resulted in the upregulation of ROS and DNA damage to enhance cancer progression 194. Overexpression of HO-1 resulted in the upregulation of proangiogenic enzyme thymidine phosphorylase, which significantly elevates angiogenesis in cancer cells, but in *in vitro*, it suppressed the proliferation and migration of cancer cells 195. Therefore, more research is required to determine the precise role of HO-1 in diverse stress conditions in cancer.

#### 7.3.2 Targeting HO-1 in cancer

In cancer progression, HO-1 is remarkably overexpressed in different types of cancer and can negatively affect the treatment outcome, particularly with its upregulation in chemotherapy and photodynamic therapy 196. HO-1 may act as an anti-apoptotic defense against tumors, protecting tumor cells from the oxidative stress produced by excessive nitric oxide produced during the growth of solid tumors. Thus, the HO-1 inhibitor zinc protoporphyrin (ZnPP) significantly reduced tumor growth in a mouse model 197. Poly (ethylene glycol)-conjugated ZnPP (PEG-ZnPP) has been proposed for use in solid tumor cancers because it has tumor-selective HO-1 inhibitory activity and increases the apoptosis of cancer cells 198. The increased expression of HO-1 in PC cell lines treated with gemcitabine or radiation has been associated with resistance to chemotherapy and radiation therapy. Thus, inhibition of HO-1 expression increases tumor cells' sensitivity to radiation therapy and chemotherapy, thereby increasing their efficacy against PC cells 199. Similarly, the inhibition of HO-1 or direct interference with HO-1 metabolites may be a new strategy for PC treatment, as HO-1 inhibition or iron chelation in human PC cell lines has increased the sensitivity and susceptibility to chemotherapy in an *in vivo* model 200. The expression of HO-1 protein was higher in colon cancer cell line FHC compared to normal tissues by 1.5-fold as well as the HO-1 mRNA and enzymatic activity. Therefore, the viability of colon cancer cells declined when treated with zinc protoporphyrin, an inhibitor of HO-1, indicating that HO-1 is a valuable biomarker for anti-colon cancer therapy 201. A series of novel indolyl-chalcone derivatives were synthesized and evaluated for anticancer activity by demonstrating the effect of *in vitro* and *in vivo* cell apoptosis of a human lung cancer cell line A549 by activating the Nrf2/HO-1 pathway without any change in normal angiogenesis 202. The sensitivity to chemotherapy was increased when SLV-1199, a pharmacological inhibitor of HO-1 activity, was used in PANC-1 and DU-145 cell lines 203. Demonstration of the Nrf2/HO-1 axis may be an important target for breast cancer treatment, as overexpression of miR-140-5p in hypoxic conditions attenuates the progression of hypoxia-induced breast cancer by reducing the expression of Nrf2 204. Collectively, HO-1 can be used as an important therapeutic target in cancer therapy, which may provide a novel strategy to increase the therapeutic efficacy of chemotherapy and radiation therapy.

## 8. Crosstalk between Nrf2, Sestrin2 and HO-1 in oxidative stress

During oxidative stress, various endogenous antioxidant proteins, and vitagene-originated proteins, interact in a hermetic manner to produce the best possible outcome for cells 63. At the initial stage of oxidative stress, Nrf2 is activated and translocated into the nucleus to bind with Antioxidant Response Elements (AREs). The activation of AREs triggers the upregulation of various antioxidant genes, including HO-1. HO-1 can break down heme, a pro-oxidant molecule, into biliverdin, carbon monoxide, and iron, which have antioxidant and anti-inflammatory properties, thereby reducing oxidative stress and protecting cells from damage 32. Additionally, the expression of Sestrin family proteins can be regulated by HO-1 by promoting its degradation.

Sestrin2 is a stress-responsive protein that regulates various cellular functions, such as antioxidant defense, autophagy, and mTOR signaling 205. Recent studies suggest Sestrin2 may regulate HO-1 expression and activity in stressed conditions. It was found that Sestrin2 deficiency led to a reduction in HO-1 expression and increased susceptibility to oxidative stress-induced cell death in cardiomyocytes. A research study has elucidated the prominent involvement of Sestrin2 as a fundamental constituent within the context of major depressive disorder, thereby illuminating its expansive implications across a spectrum of diverse pathological conditions 206. The precise mechanism underlying the crosstalk between Sestrin 2, and HO-1 is undetermined, although it may involve signaling pathways such as the Nrf2 pathway. Sestrin2 has been shown to stimulate Nrf2 signaling and enhance the expression of Nrf2 target genes such as HO-1, which may contribute to its antioxidant effects 207.

Overall, in stressed settings, the interaction between Nrf2, Sestrin2, and HO-1 may regulate cellular antioxidant defense mechanisms and protect cells from oxidative damage. However, the crosstalk and interaction between these proteins are complex and context-specific, and more studies are needed to fully understand the mechanism underlying this crosstalk and its consequences for cellular stress response.

## 9. Conclusion and future perspectives

Generally, it is widely known that oxidative stress is highly correlated with the incidence of metabolic diseases and cancer. This review examined the results from the latest research on antioxidant genes that can overcome metabolic diseases and cancers (Table 1). These recent studies may advance our biological understanding of the role of antioxidant genes in human disease. Nrf2 and Nrf2-regulated genes, HO-1, and Sestrin, positively affect metabolic diseases through their antioxidant activities or by controlling other metabolic signals. However, there are many 'grey areas' in understanding the dual roles of Nrf2, Sestrin, and HO-1 in cancer, with some studies revealing positive effects and others suggesting negative effects. This ambiguity indicates that further studies are needed to unravel these genes' complex relationship with various disease states. Despite remaining uncertainties, a detailed compilation of the results of various pathological models, along with discovering compounds that trigger antioxidant-mediated signaling pathways, will lead to the discovery of new targets for treating metabolic diseases and cancer.

## Figures and Tables

**Table 1:** Role of antioxidant genes Nrf2, Sestrin2 and HO-1 in metabolic disease and cancer

Gene	Involved Metabolic Diseases and Regulatory Mechanisms	Involved Cancer and Regulatory Mechanisms	Reference
Nrf2	**Obesity**: AMPK activation, reduction of lipogenesis, enhancement of β-oxidation, insulin sensitivity improvement **Diabetes**: Insulin sensitivity improvement, protection of pancreatic beta cells, reduction of oxidative stress, AMPK activation **Non-alcoholic Steatohepatitis (NASH)**: Reduction of lipogenesis, enhancement of β-oxidation, AMPK activation	Colorectal Cancer: Chemoresistance through Nrf2 activation via KEAP1 and CUL3 mutations Breast Cancer: Chemoresistance via constitutive Nrf2 upregulation, leading to increased antioxidant gene expression Pancreatic Cancer: Chemoresistance enhancement, tumor progression through Nrf2 interaction with ATDC Gastric Cancer: Chemoresistance via Nrf2 activation and antioxidant gene upregulation Cervical Cancer: Increased chemosensitivity and suppression of tumor growth through Nrf2 inhibition or knockout Glioma: Tumor progression and poor prognosis through Nrf2 activation, suppression of tumorigenesis through Nrf2 knockout Non-Small Cell Lung Cancer (NSCLC): Tumor progression and treatment resistance through Nrf2 activation and interaction with PAQR4	26, 27, 70, 71, 78, 85, 89, 93, 95, 96, 97, 98
Sestrin2	**Obesity**: AMPK activation, inhibition of mTORC1 activity, enhancement of autophagy **Diabetes**: AMPK activation, improvement of insulin sensitivity, reduction of oxidative stress, inhibition of mTORC1 activity **Insulin Resistance**: AMPK activation, enhancement of autophagy, inhibition of mTORC1 activity **Liver Fat Accumulation**: Inhibition of Liver X receptor α, prevention of liver fat accumulation	**Endometrial Cancer**: mTORC1 inhibition, suppression of cancer cell proliferation, induction of autophagy **Colorectal Cancer**: mTORC1 inhibition, AMPK suppression, induction of apoptosis, reduction of cancer cell viability **Lung Cancer**: AKT/mTOR/P70S6K signaling suppression, inhibition of proliferation and migration of cancer cell **Breast Cancer**: mTORC1 inhibition, suppression of cancer progression, induction of apoptosis **Hepatocellular Carcinoma**: p53-dependent mechanisms, suppression of cancer progression, induction of autophagy **Bone Cancer**: mTORC1 inhibition, suppression of cancer cell proliferation, induction of autophagy **Pancreatic Cancer**: Inhibition of cancer cell migration, proliferation, and invasion through Nrf2/KEAP1/HO-1/NQO-1 signaling **Bladder Cancer**: Autophagy modulation, induction of apoptosis, suppression of cancer progression **Cervical Cancer**: Autophagy modulation, suppression of cancer progression, induction of apoptosis **Osteosarcoma**: mTOR inhibition through p-ERK-eIF2α-CHOP pathway, suppression of apoptosis, induction of chemoresistance **Melanoma**: Enhancement of cancer cell survival, promotion of chemoresistance, activation of AKT pathway	28, 29, 80, 131, 132, 133, 134, 137, 138, 139, 140, 141, 143, 144, 149, 150, 157
HO-1	**Obesity**: Maintenance of redox homeostasis, inhibition of adipogenesis, regulation of Peg1/Mest and Wnt10b/β-catenin/Sonic hedgehog signaling pathways **Diabetes**: Protection of pancreatic beta cells, reduction of oxidative stress, induction of glucose metabolism-related genes (e.g., GLUT4, adiponectin), activation of AMPK signaling **Insulin Resistance**: Improvement of insulin sensitivity, reduction of pro-inflammatory cytokines, activation of the HO-1-adiponectin axis, and AMPK signaling	**Melanoma**: Promotion of tumor aggressiveness, increased viability, proliferation, and metastasis **Acute Myeloid Leukemia**: Chemoresistance through HO-1 overexpression, reduced sensitivity to cytarabine and daunorubicin **Multiple Myeloma**: Induction of IL-6, increased resistance to lenalidomide treatment, survival factor promotion **Lung Cancer**: Promotion of lung metastasis, activation of VEGF and IL-10 **Colon Cancer**: Modulation of Nrf2/HO-1 signaling, upregulation of vimentin, downregulation of E-cadherin, suppression of immune responses **Pancreatic Cancer**: Activation of the Sonic Hedgehog (SHH) signaling pathway, promotion of cell proliferation **Prostate Cancer**: Controversial role; prevention of angiogenesis, progression of the tumor **Breast Cancer**: Dual role; promotion or suppression of tumor proliferation depending on angiogenesis and subcellular localization **Head and Neck Cancer**: Enhancement of cancer cell growth, pro-tumor activity enhancement	161, 164, 165, 166, 167, 168, 170, 179, 181, 182, 183, 184, 185, 186, 187, 188, 190, 199
